# Dietary Scale in a Poor-Law Infirmary

**Published:** 1920-10-30

**Authors:** 


### Dietary Scale in a Poor-law Infirmary.

Camberwell Board of Guardians have adopted a new dietary table for the patients in their infirmary. There are six grades of meals to cover the various conditions of the patients. All are ample, especially No. 1 (full) grade, which is as follows:—

Breakfast.

| | Males. | Females. |
|---|---|---|
| Bread | 6 oz. | 5 oz. |
| Butter | ½ oz. | ½ oz. |
| Tea | 1 pint | 1 pint |

Dinner.

| | Males. | Females. |
|---|---|---|
| Sunday, roast mutton | 6 oz. | 4 oz. |
| Monday, roast beef | 6 oz. | 4 oz. |
| Tuesday, bacon | 6 oz. | 4 oz. |
| Wednesday, boiled or roast mutton | 6 oz. | 4 oz. |
| Thursday, stew | 1 pint | 1 pint |
| Friday, fish | 8 oz. | 8 oz. |
| Saturday, roast beef | 6 oz. | 4 oz. |
| Potatoes, daily | 8 oz. | 6 oz. |
| Bread, daily | 3 oz. | 3 oz. |

Green vegetables (varied in season).

Tea consists of the same articles of diet and the same quantities as are provided for breakfast.

No. 2 (fish) diet affects the dinner routine only. Eight ounces are provided for both men and women. On Mondays, Wednesdays, and Saturdays it is fried, and on the remaining days of the week it is boiled.

Particulars are provided of how to prepare some of the articles of diet. For instance, to make a gallon of tea the instructions are: Tea, 2 oz.; boiling water, 8 pints; sugar, 5 oz. (when possible).

The recipe for making beef-tea is as follows: "Half a pound of shin of beef, free from fat and bone, cut finely; add one quart of water, and simmer to a pint; the temperature is not to exceed 180 degs. Fahr.; add one pound of Oxo, or equivalent, to every 22 pints."

Stew is to be made according to the following: "Forty-eight oz. of meat to each gallon of liquor of boiled meat, with addition to mutton bones; 8 oz. of barley; onions, 8 oz.; carrots, 8 oz.; and other vegetables and herbs in season. During the stewing temperature is not to exceed 180 degs. Fahr."

Barley-water is to be prepared by providing 2 oz. of barley to every pint of water, and the recipe for jelly is as follows: "Gelatine (Nelson's), 1½ oz.; sugar, 6 oz.; citra acid to flavour; add water to make 1 quart"

